# Autism spectrum disorders - gender differences and the diagnosis dilemma

**DOI:** 10.1192/j.eurpsy.2023.1523

**Published:** 2023-07-19

**Authors:** J. D. C. Moura, J. Leal, J. F. Cunha, D. Seabra, S. Torres, T. Rocha, I. Lopes, B. Barata

**Affiliations:** Departamento de Psiquiatria e Saúde Mental, Centro Hospitalar Barreiro-Montijo, Lisboa, Portugal

## Abstract

**Introduction:**

Autism Spectrum Disorder (ASD) is a neurodevelopmental disorder characterized by social and communication deficits and restricted and repetitive or stereotyped behaviours. The prevalence of ASD has been thought to be higher in men, which may reflect aspects of the own aetiology of the disorder. Still, it may also be associated with misdiagnosis or missed diagnosis of females with autism due to specific phenotypic traits.

**Objectives:**

To explore the differences between sex/gender in autism’s clinical presentation.

**Methods:**

Non-systematic literature review using the most relevant papers found on PubMed and Google Scholar using the following keywords: “autism spectrum disorder”, “gender differences”, and “autistic women”.

**Results:**

Autistic women seem to have a “camouflage” phenomenon, characterized by a high level of functioning, less unusual play or restricted interests, better socio-emotional reciprocity and coping behaviours. Therefore, women with ASD commonly have an anteriority of multiple diagnoses, which delays their access to the support and care they need.

**Conclusions:**

Professionals must be aware of the sex/gender clinical differences to prevent the misdiagnosis or missed diagnosis of females with autism. Moreover, the current clinical criteria used to diagnose ASD may underserve the female population and deserve to be reviewed.

**Disclosure of Interest:**

None Declared

